# Supplementary material for: An atlas of small non-coding RNAs in human preimplantation development
Source: Nat Commun. 2024 Oct 5;15:8634. doi: 10.1038/s41467-024-52943-w (PMC11452719; doi:10.1038/s41467-024-52943-w)
Supplement: Supplementary file 1 — Supplementary Information [file 41467_2024_52943_MOESM1_ESM.pdf]

An atlas of small non-coding RNAs in Human Preimplantation Development

Stewart J. Russell[1†], Cheng Zhao[2,3†], Savana Biondic[4,5], Karen Menezes[1], Michael Hagemann-Jensen[6], Clifford L. Librach[1,7,8,9,10,12], Sophie Petropoulos[2,3,4,5,6,11*]

1 CReATe Fertility Centre, Toronto, ON, Canada

2 Department of Clinical Science, Intervention and Technology, Karolinska Institutet, Stockholm, Sweden

3 Division of Obstetrics and Gynecology, Karolinska Universitetssjukhuset, Stockholm, Sweden

4 Faculty of Medicine, Molecular Biology Program, Université de Montréal, Montréal, QC, Canada

5 Centre de Recherche du Centre Hospitalier de l'Université de Montréal, Axe Immunopathologie, Montréal, Canada

6 Department of Cell and Molecular Biology, Karolinska Institutet, 171 77 Stockholm Sweden

7 Department of Laboratory Medicine and Pathobiology, University of Toronto, Toronto, ON, Canada

8 Department of Obstetrics and Gynaecology, University of Toronto, Toronto, ON, Canada

9 Department of Physiology, University of Toronto, ON, Canada

10 Sunnybrook Research Institute, Toronto, ON, Canada

11 Faculty of Medicine, Département de Médecine, Université de Montréal, Montréal Canada

12 Institute of Medical Sciences, University of Toronto, Toronto, ON, Canada

† These authors contributed equally

*corresponding author: sophie.petropoulos@umontreal.ca, sophie.petropoulos@ki.se

Supplementary Information

Included are 14 Supplementary Figures (including legends) and 1 Supplementary Table

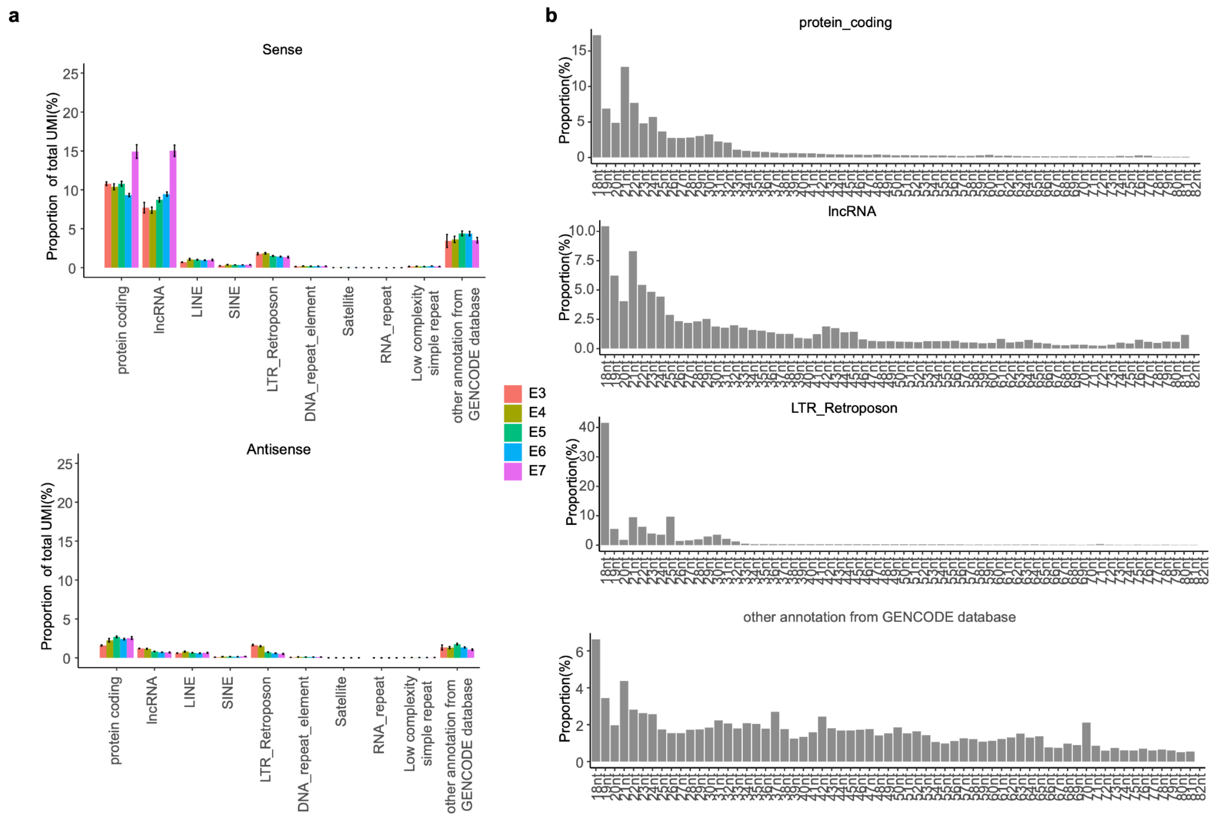

Supplementary Fig. 1 Non-canonical small RNA mapping a Proportion of UMIs overlapping existing annotations from GENCODE and Repbase by day of embryonic development stratified by sense and antisense of annotation (E - embryonic day). Error bars represent standard error of the mean (SEM) values. **b** Length distribution of UMIs overlapping with protein-coding genes, long non-coding (lnc)RNA, long terminal repeat (LTR) retroposons, and other grouped annotations from GENCODE. Embryo and cell numbers are presented in Fig 1a.

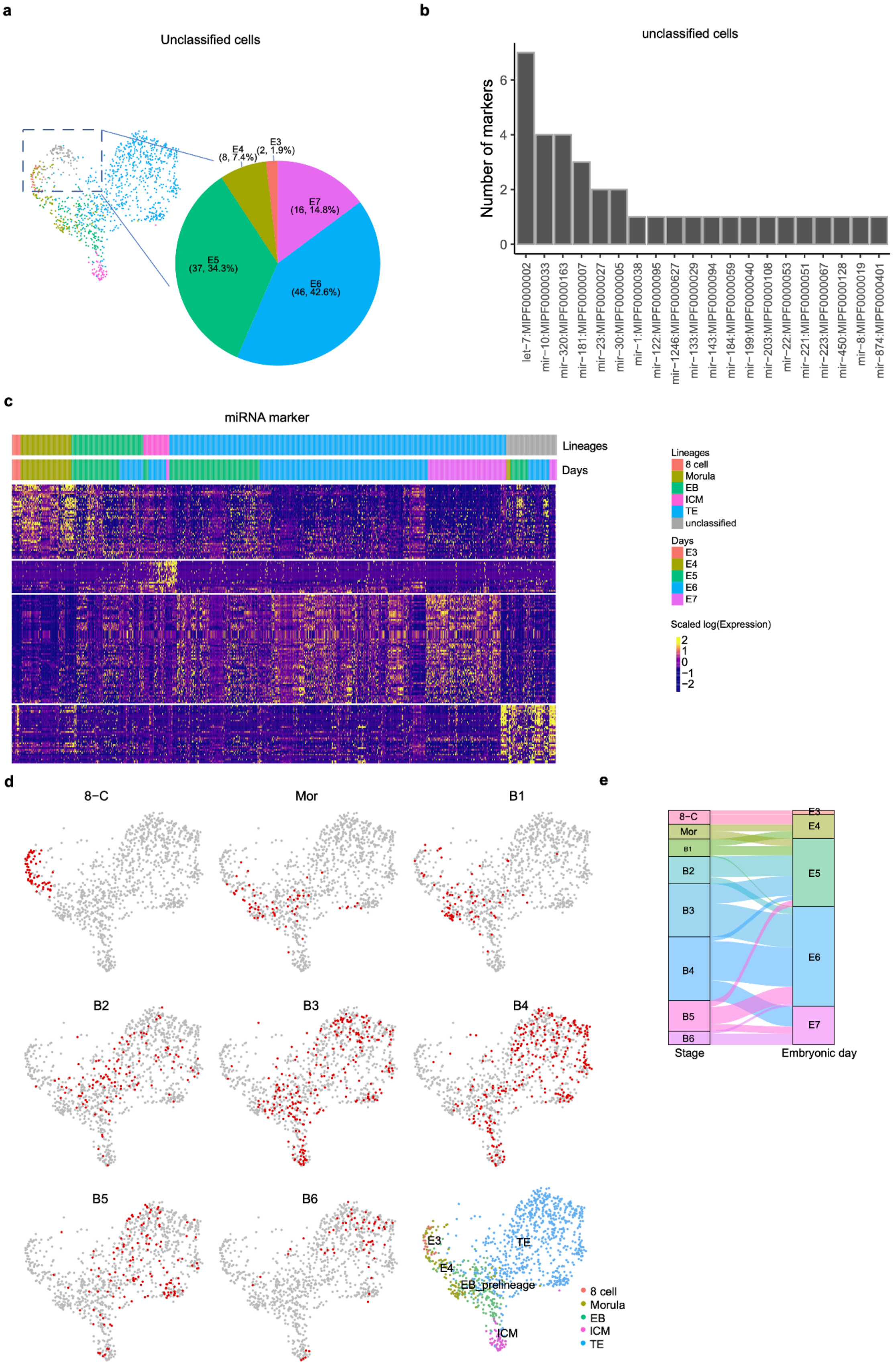
a
Unclassified cells
E3 (2, 1.9%)
E4 (8, 7.4%)
E7 (16, 14.8%)
E5 (37, 34.3%)
E6 (46, 42.6%)
b
unclassified cells
Number of markers
let−7:MIPF0000002
mir−10:MIPF0000033
mir−320:MIPF0000163
mir−181:MIPF0000007
mir−23:MIPF0000027
mir−30:MIPF0000005
mir−1:MIPF0000038
mir−122:MIPF0000095
mir−1246:MIPF0000627
mir−133:MIPF0000029
mir−143:MIPF0000094
mir−184:MIPF0000059
mir−199:MIPF0000040
mir−203:MIPF0000108
mir−22:MIPF0000053
mir−221:MIPF0000051
mir−223:MIPF0000067
mir−450:MIPF0000128
mir−8:MIPF0000019
mir−874:MIPF0000401
c
miRNA marker
Lineages
Days
Lineages
8 cell
Morula
EB
ICM
TE
unclassified
Days
E3
E4
E5
E6
E7
Scaled log(Expression)
d
8−C
Mor
B1
B2
B3
B4
B5
B6
E3
E4
TE
EB_prelineage
ICM
8 cell
Morula
EB
ICM
TE
e
8−C
Mor
B1
B2
B3
B4
B5
B6
Stage
E3
E4
E5
E6
E7
Embryonic day

Supplementary Fig. 2: miRNA profiling of unclassified cells. **a** Proportion of unclassified cells by embryonic day. **b** The proportion of miRNA markers for unclassified cells in miRNA families. **c** Heatmap of marker miRNA expression in all cell types. **d** Highlighting cells from different embryonic stages on the human embryonic reference. **e** The alluvial plot compares embryonic stages and embryonic days. E - embryonic day; ICM - inner cell mass; TE - trophectoderm; EB - early blastocyst; 8-C - 8 cell; Mor - morula; B - blastocyst stage; (embryo staging from Meistermann et al. 2021 was used).

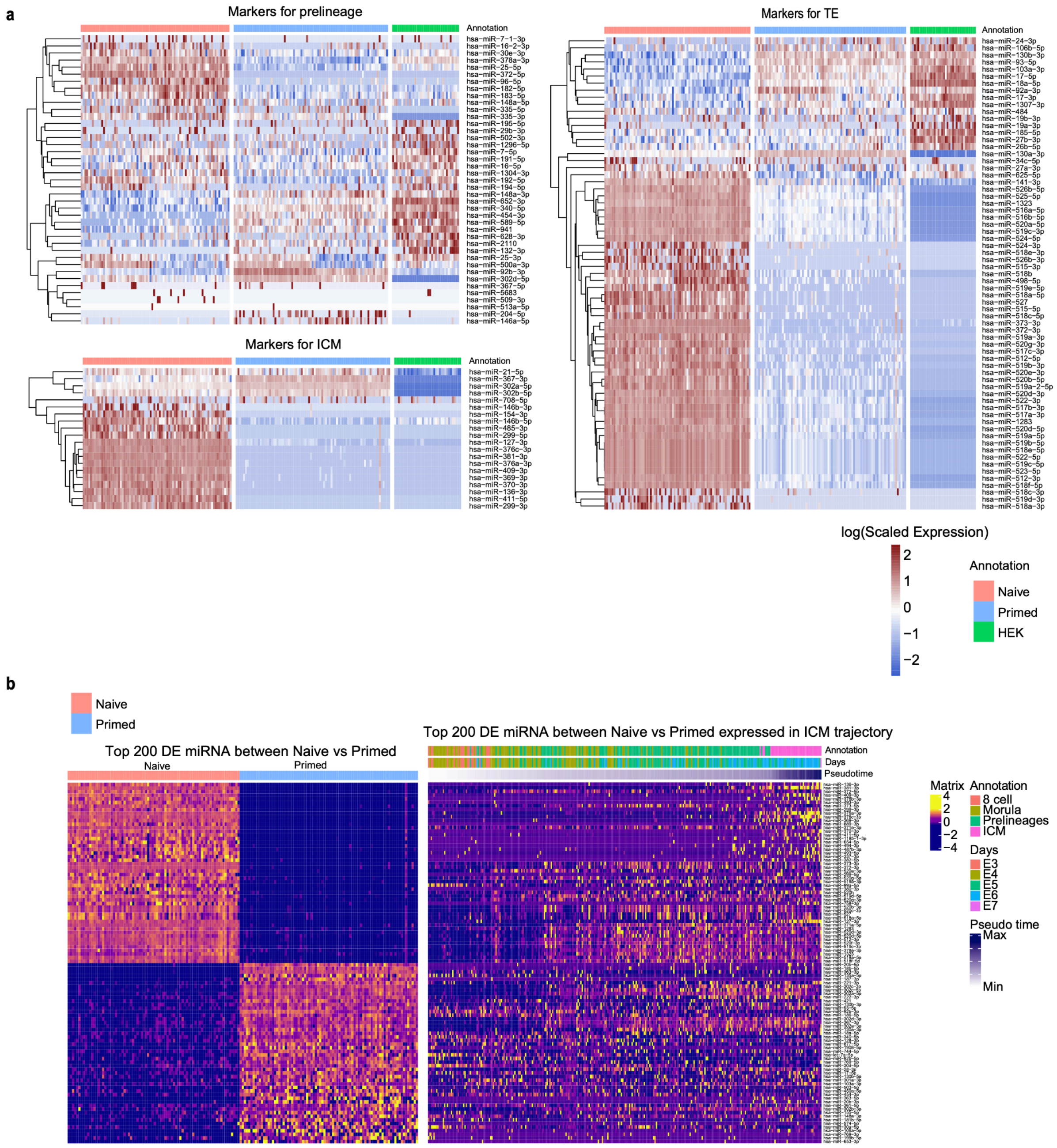

Supplementary Fig. 3 Lineage-associated miRNA expression in stem cells. a Heatmaps of lineage-specific miRNA expression in naïve, primed ESCs and HEK293 cells. **b** Heatmaps of top 200 naive vs primed ESC markers (left) and expression of these markers in ICM pseudotime trajectory (right). E - embryonic day; ICM - inner cell mass; EB - early blastocyst; HEK - human embryonic kidney cells.

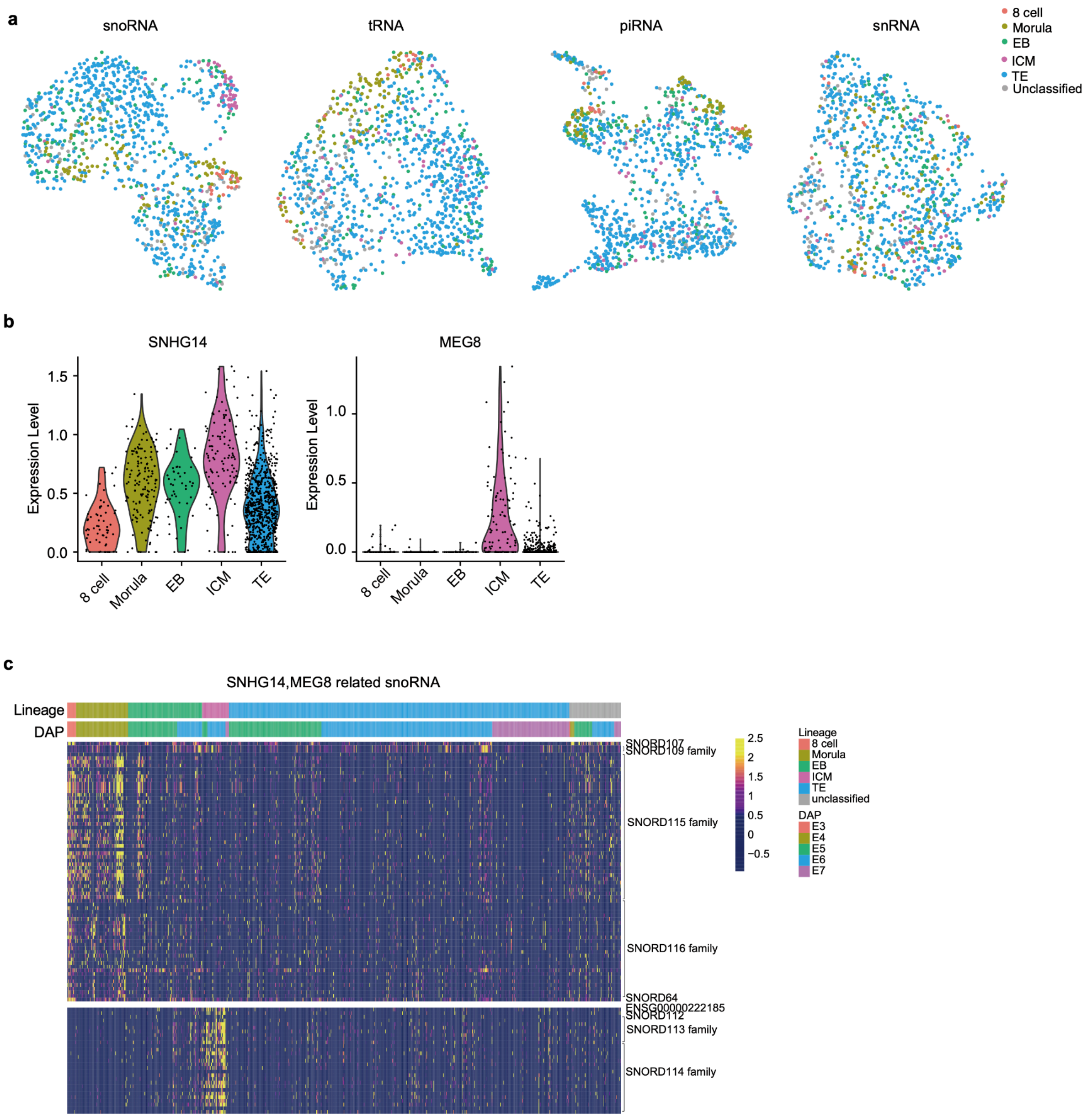

Supplementary Fig. 4: SnoRNA are developmentally regulated in peri-implantation development. **a** UMAP of snoRNA, tRNA, piRNA, and snRNA expression coloured by lineage. **b** SnoRNA precursor genes, SNHG14 and MEG8 expression across lineages (Petropoulos et al. 2016). **c** Heatmap of SNHG14 and MEG8 related snoRNA expression by SNORD family and embryonic lineage. E - embryonic day; ICM - inner cell mass; TE - trophectoderm; EB - early blastocyst.

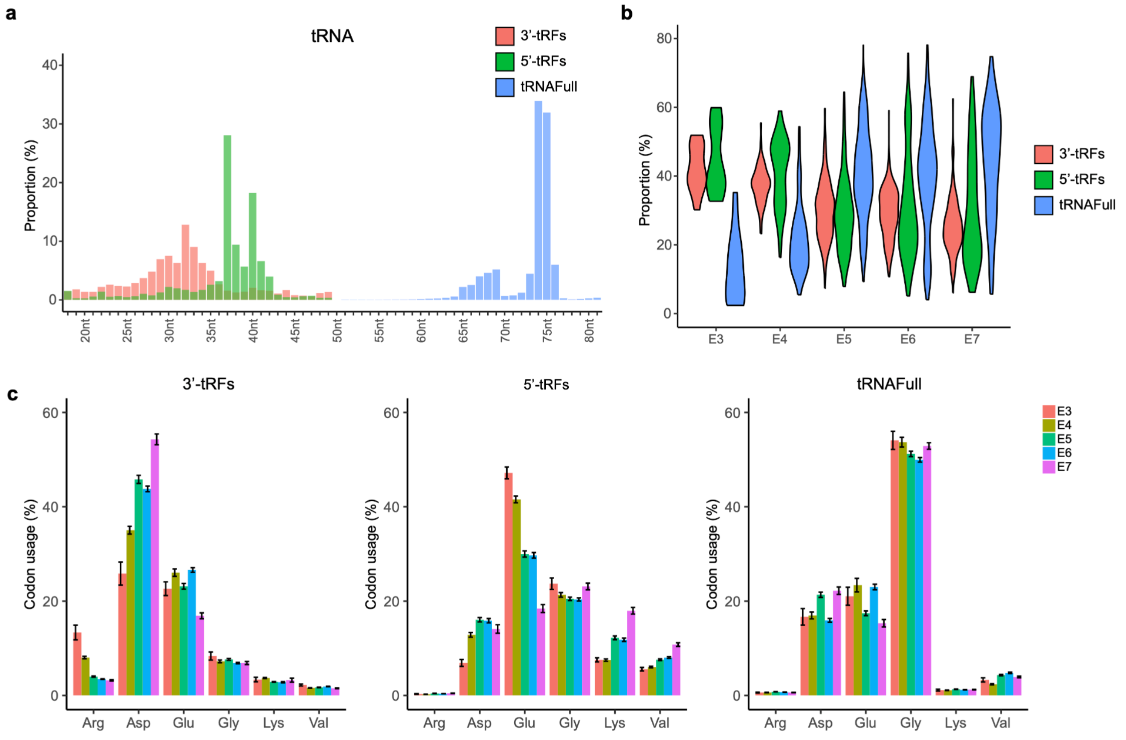

Supplementary Fig.5: tRNA profiling. **a** Length distributions of 5'-tRFs, 3'-tRFs, and full length tRNAs in E3 to E7 embryos. **b** Relative proportions of tRNA halves and full length tRNAs by embryonic day (E). **c** Codon usage proportions by tRNA codon type and embryonic day. Embryo and cell numbers are presented in Fig 1a. Error bars represent standard error of the mean (SEM) values.

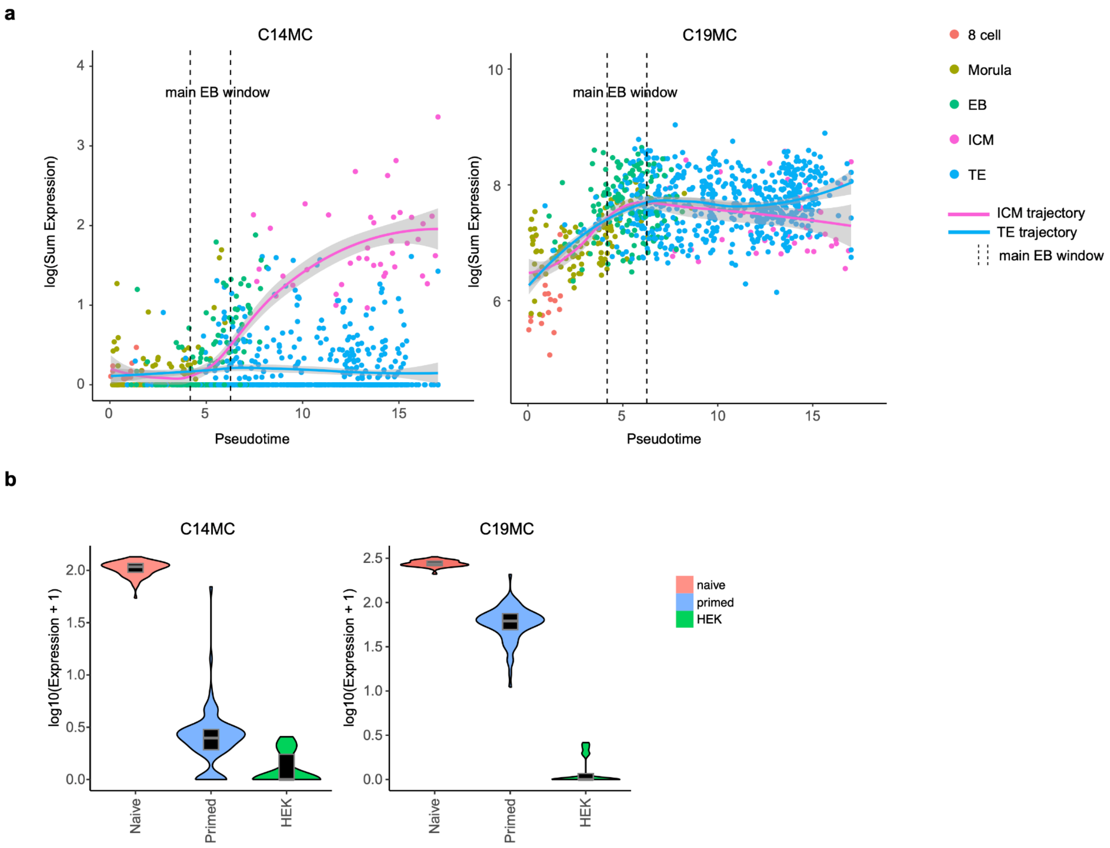

Supplementary Fig. 6: Expression of C14MC and C19MC across (**a**) embryonic pseudotime and (**b**) in ESCs. ICM - inner cell mass; TE - trophectoderm; EB - early blastocyst. The confidence interval (error bands, 95%) is indicated by bandwidth. The measure of center and confidence intervals were calculated using the "loess" function with default parameters in R software. The boxplot rectangles represent the first and third quartiles, a vertical line inside the box indicates the median value.

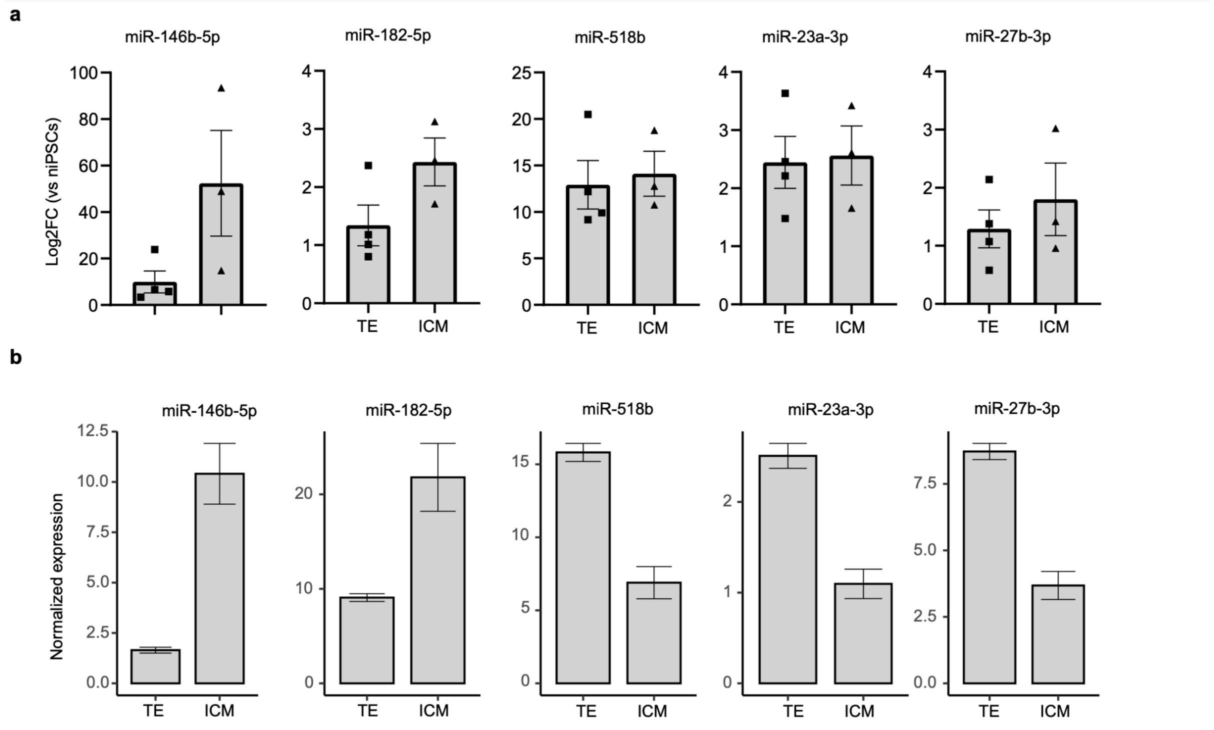

Supplementary Fig. 7: a RT-qPCR of selected miRNAs in trophectoderm (TE) and inner cell mass (ICM) human embryo biopsies. Congruent with our Small-seq data, miR-146b-5p and miR-182-5p were enriched in the ICM. In contrast, miR-518b, miR23a-3p and miR-27b-3p were enriched in the TE according to the Small-seq data, but differences were not detected by RT-qPCR. Data were normalised to niPSC, n=3 embryos (ICM) and n=4 embryos (TE). **b** miRNA expression from E6 and E7 embryo based on small-seq data, n= 31 cells (ICM) and n=410 cells (TE). Error bars represent standard error of the mean (SEM) values.

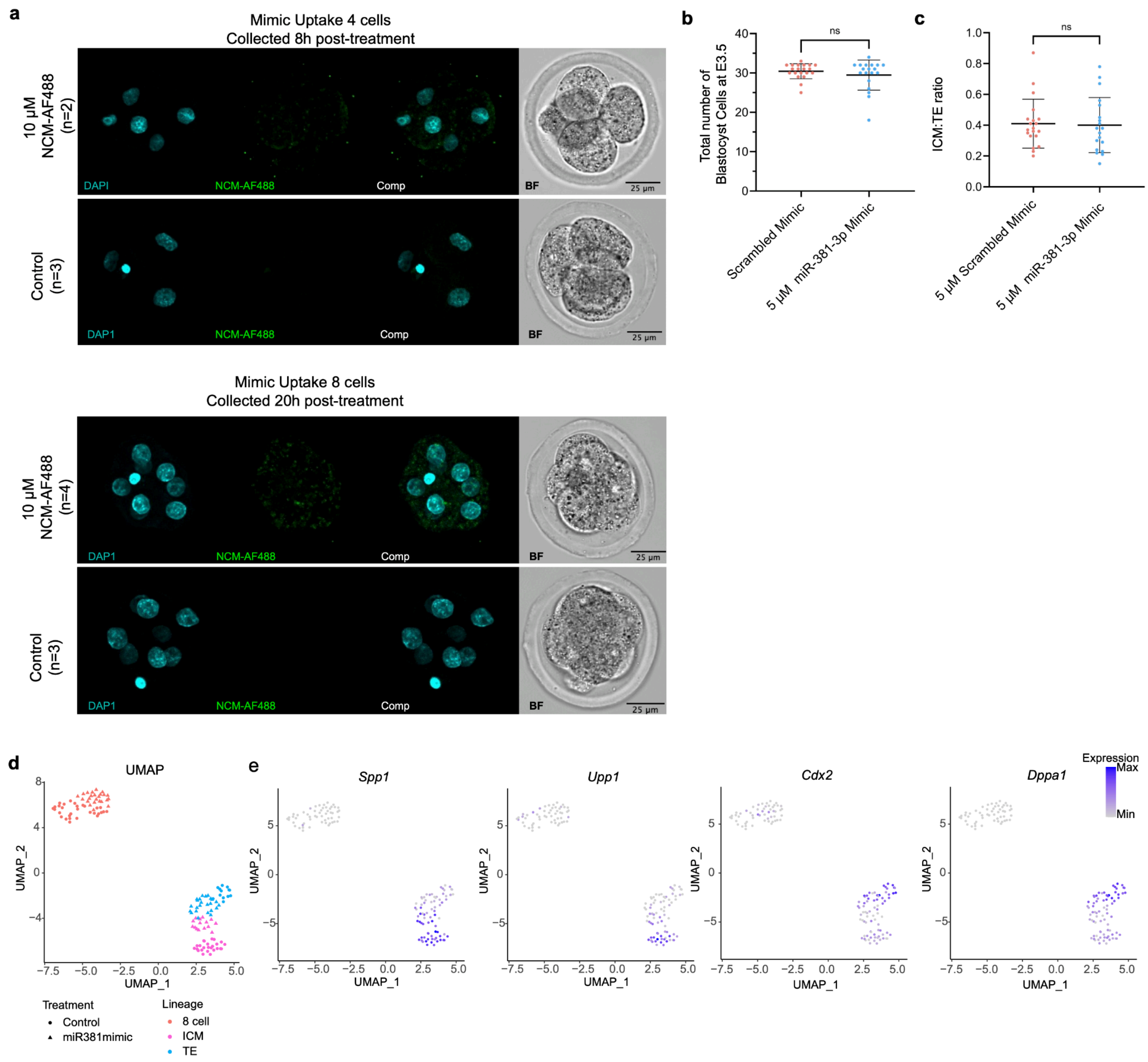

Supplementary Fig. 8 miRNA mimic treatment in mouse embryos. a Representative images of mouse embryos cultured with (10 µM NCM-AF488) or without (Control) the scrambled negative control mimic (NCM) conjugated to Alexa Fluor (AF) 488 for 8h (from 2-cell to 4-cell stage) and 20h (from 2-cell to 8-cell stage). **b** Average number of embryonic cells in control versus 5 µM miR-381-3p mimic (n=20 control embryos, pink and n=19 mimic embryos, blue) -treated blastocysts, where each datapoint represents the total number of cells in one embryo. **c** Average ICM:TE Ratio in control versus 5 µM miR-381-3p mimic (n=20 control embryos, pink and n=19 mimic embryos, blue)-treated blastocysts, where each data point represents the ICM:TE ratio of one embryo. **d** Two-dimensional UMAP visualisation of single-cell transcriptomes of control and miR-381-3p mimic-treated mouse

embryos cells, coloured by cell identities and shaped by different treatments. **e** UMAP plot of cells showing selected marker gene expression. E - embryonic day; ICM - inner cell mass; TE - trophectoderm.

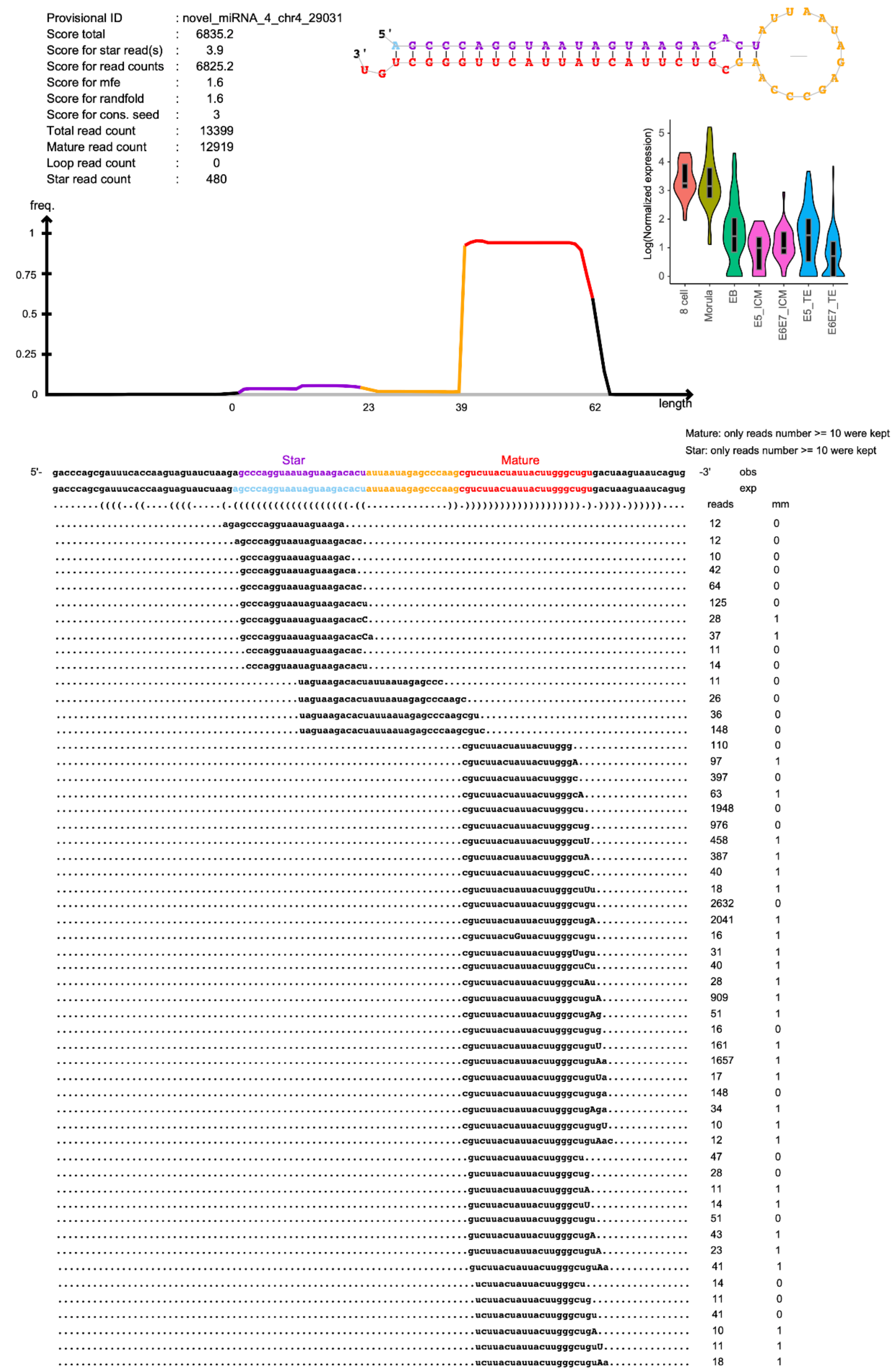

Supplementary Fig. 9 miRDeep2 output and human embryonic expression for novel_miRNA_4_chr4_29031. E - embryonic day; ICM - inner cell mass; TE - trophectoderm; EB - early blastocyst.

Provisional ID	:	novel_miRNA_4_chr15_13546 (hsa-miR-1839-5p)
Score total	:	229.9
Score for star read(s)	:	3.9
Score for read counts	:	222.4
Score for mfe	:	-0.9
Score for randfold	:	1.6
Score for cons. seed	:	3
Total read count	:	448
Mature read count	:	420
Loop read count	:	0
Star read count	:	28

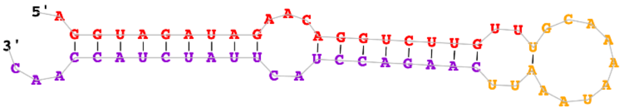

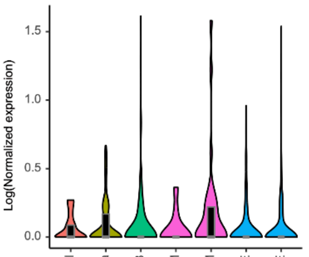

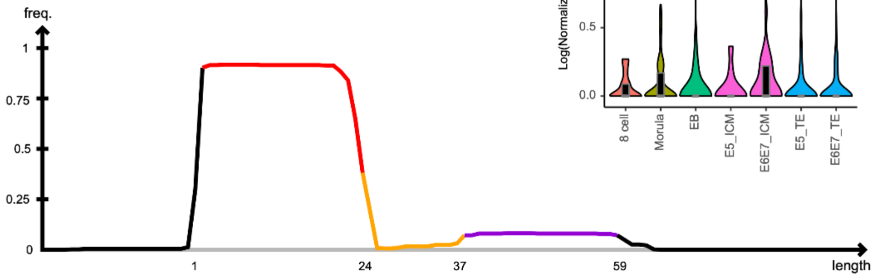


```
                         Mature                                   Star
5'- uuuuagccuccuaaaugaaaagguagauagaacaggucuuguuugcaaaauaaaauucaagaccuacuuaucuaccaacagcaauuacacgcuuuccauugucuuccuauag -3'  obs
    uuuuagccuccuaaaugaaaagguagauagaacaggucuuguuugcaaaauaaaauucaagaccuacuuaucuaccaacagcaauuacacgcuuuccauugucuuccuauag       exp
    ...(((.......((((....(((((((((...((((((((..(..........)..))))))))..)))))))))...(((........)))...))))......)))...       reads  mm
    ..uuagccuccuaaaugaaaagguagauagaacaggu...........................................................................       1      0
    ....agccuccuaaaugaaaagguagau....................................................................................       1      0
    ..................aaagguagauagaacaggucuu........................................................................       1      0
    ..................aaagguagauagaacaggucuug.......................................................................       3      0
    ...................aagguagauagaacaggucu.........................................................................       2      0
    ...................aagguagauagaacaggucuu........................................................................       15     0
    ...................aagUuagauagaacaggucuug.......................................................................       1      1
    ...................aagguagauagaacaggucuug.......................................................................       46     0
    ...................aagguagauagaacaggucuuU.......................................................................       2      1
    ...................aagguUgauagaacaggucuugu......................................................................       1      1
    ...................aagguagauagaacaggucuugu......................................................................       55     0
    ...................aagguagauagaacaggucuugA......................................................................       4      1
    ...................aagguaUauagaacaggucuugu......................................................................       1      1
    ...................aUgguagauagaacaggucuugu......................................................................       1      1
    ...................aagguagauagaacaggucuuguu.....................................................................       2      0
    ...................aagguagauagaacaggucuuguuu....................................................................       1      0
    ....................agguagauagaacaggucu.........................................................................       12     0
    ....................agguagauagaacaggucuu........................................................................       4      0
    ....................agguagauagaacaggucuuU.......................................................................       1      1
    ....................agguagauagaacaggucuug.......................................................................       34     0
    ....................agguagauagaacaggucuugu......................................................................       53     0
    ....................agguagauagaacaggucuugA......................................................................       6      1
    ....................agguagauagaacaggucuuguu.....................................................................       139    0
    ....................aCguagauagaacaggucuuguu.....................................................................       1      1
    ....................agguagauagaacaggucuuguuA....................................................................       13     1
    ....................agguaAauagaacaggucuuguuu....................................................................       1      1
    ....................agguagauagaacaggucuuguAu....................................................................       1      1
    ....................agguagauagaacaggucuuguuu....................................................................       12     0
    ....................agguagauagaacaggucuuguuuA...................................................................       1      1
    ....................agguagauagaacaggucuuguuugcaaaauaaaauu.......................................................       1      0
    .....................gguagauagaacaggucuug.......................................................................       1      0
    .....................gguagauagaacaggucuugu......................................................................       2      0
    .....................gguagauagaacaggucuuguu.....................................................................       2      0
    .....................gguagauagaacaggucuuguuu....................................................................       1      0
    .......................uagauagaacaggucuuguu.....................................................................       1      0
    ...........................................ugcaaaauaaaauucaagaccuac.............................................       1      0
    ............................................gcaaaauaaaauucaagaccuacuuaucuaccaacagca.............................       1      0
    ...............................................aaauaaaauucaagaccuacuuaucuaccaacagcU.............................       2      1
    ................................................aauaaaauucaagaccuacuuaucuaccaacagca.............................       2      0
    ................................................aauaaaauucaagaccuacuuaucuaccaacagcaa............................       1      0
    .....................................................aauucaagaccuacuuaucuaccaacagca.............................       3      0
    ........................................................ucaagaccuacuuaucuacc....................................       1      0
    ........................................................ucaagaccuacuuaucuaccaac.................................       2      0
    ........................................................ucaagaccuacuuaucuaccaacU................................       1      1
    .........................................................caagaccuacuuaucuaccaa..................................       3      0
    .........................................................caagaccuacuuaucuaccaaU.................................       1      1
    .........................................................caagaccuacuuaucuaccaac.................................       14     0
    .........................................................caagaccuacuuaucuaccaacU................................       1      1
    ...........................................................agaccuacuuaucuaccaaca................................       2      0
    ...........................................................agaccuacuuaucuaccaacagc..............................       2      0
    ...............................................................cuacuuaucuaccaacagca.............................       1      0
```

Supplementary Fig. 10 miRDeep2 output and human embryonic expression for

novel_miRNA_4_chr15_13546. E - embryonic day; ICM - inner cell mass; TE - trophectoderm; EB - early blastocyst.

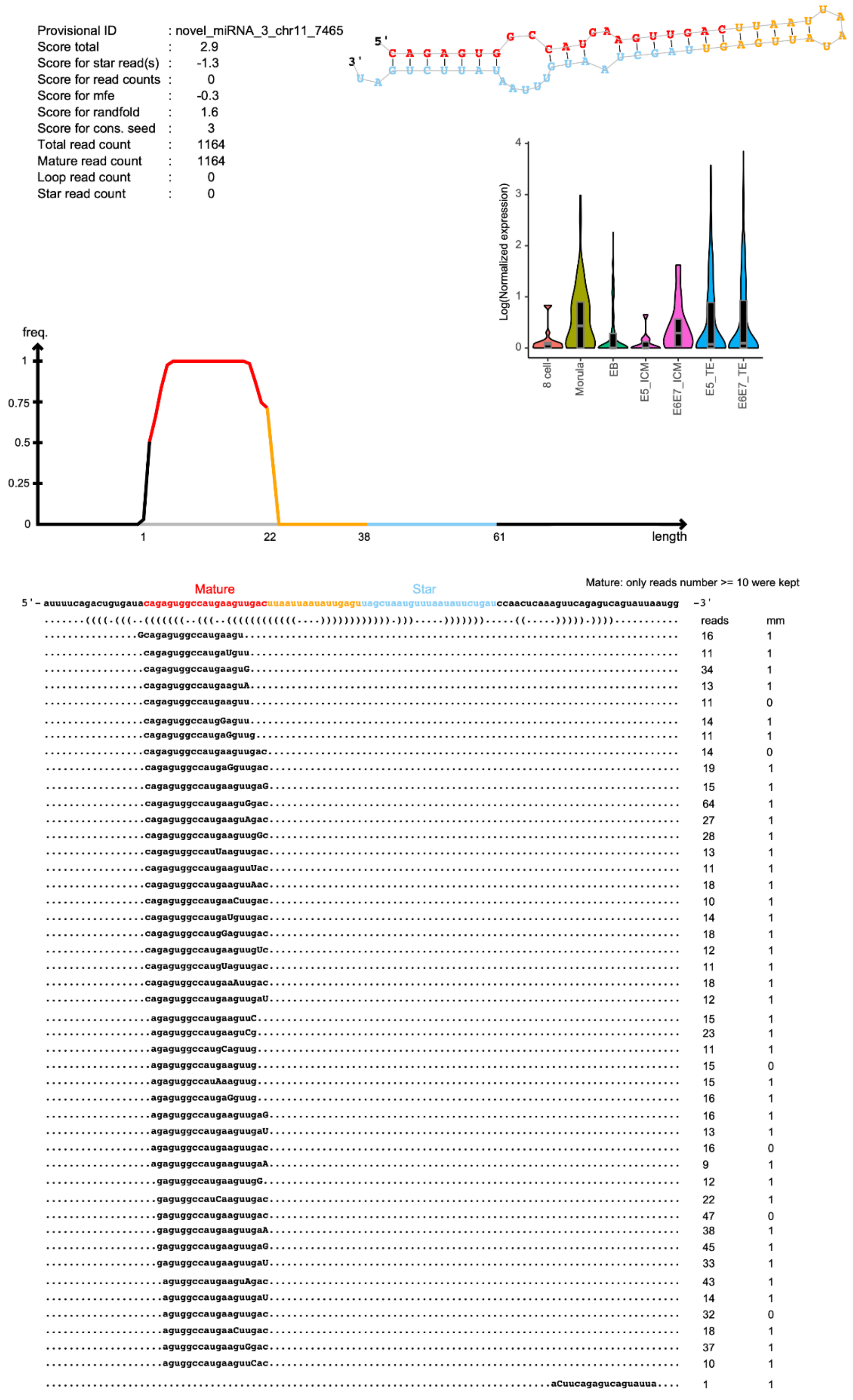

Supplementary Fig. 11 miRDeep2 output and human embryonic expression for

novel_miRNA_3_chr11_7465. E - embryonic day; ICM - inner cell mass; TE - trophectoderm; EB - early blastocyst.

a

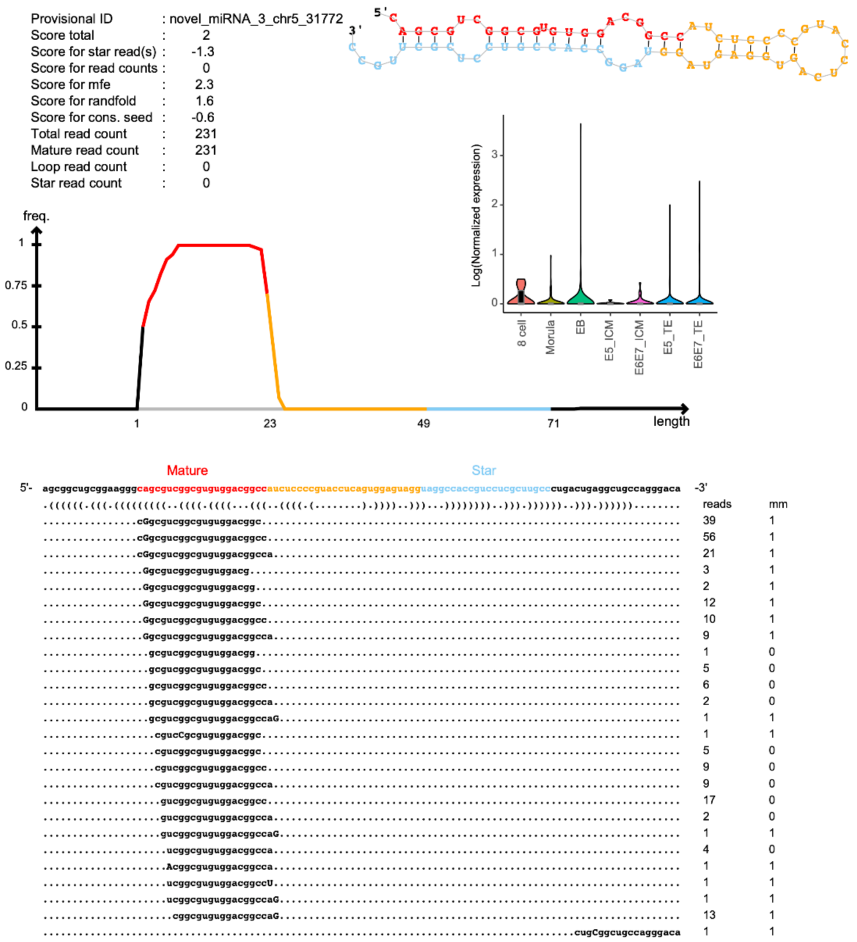

b

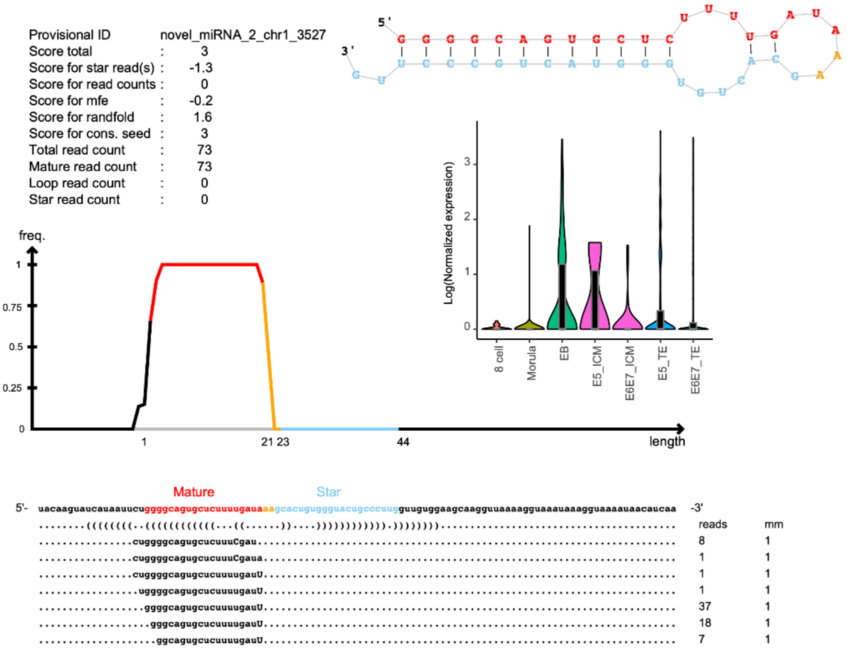

Supplementary Fig. 12 miRDeep2 output and human embryonic expression for a)

novel_miRNA_3_chr5_31772 and b) novel_miRNA_2_chr1_3527. E - embryonic day; ICM - inner cell mass; TE - trophectoderm; EB - early blastocyst.

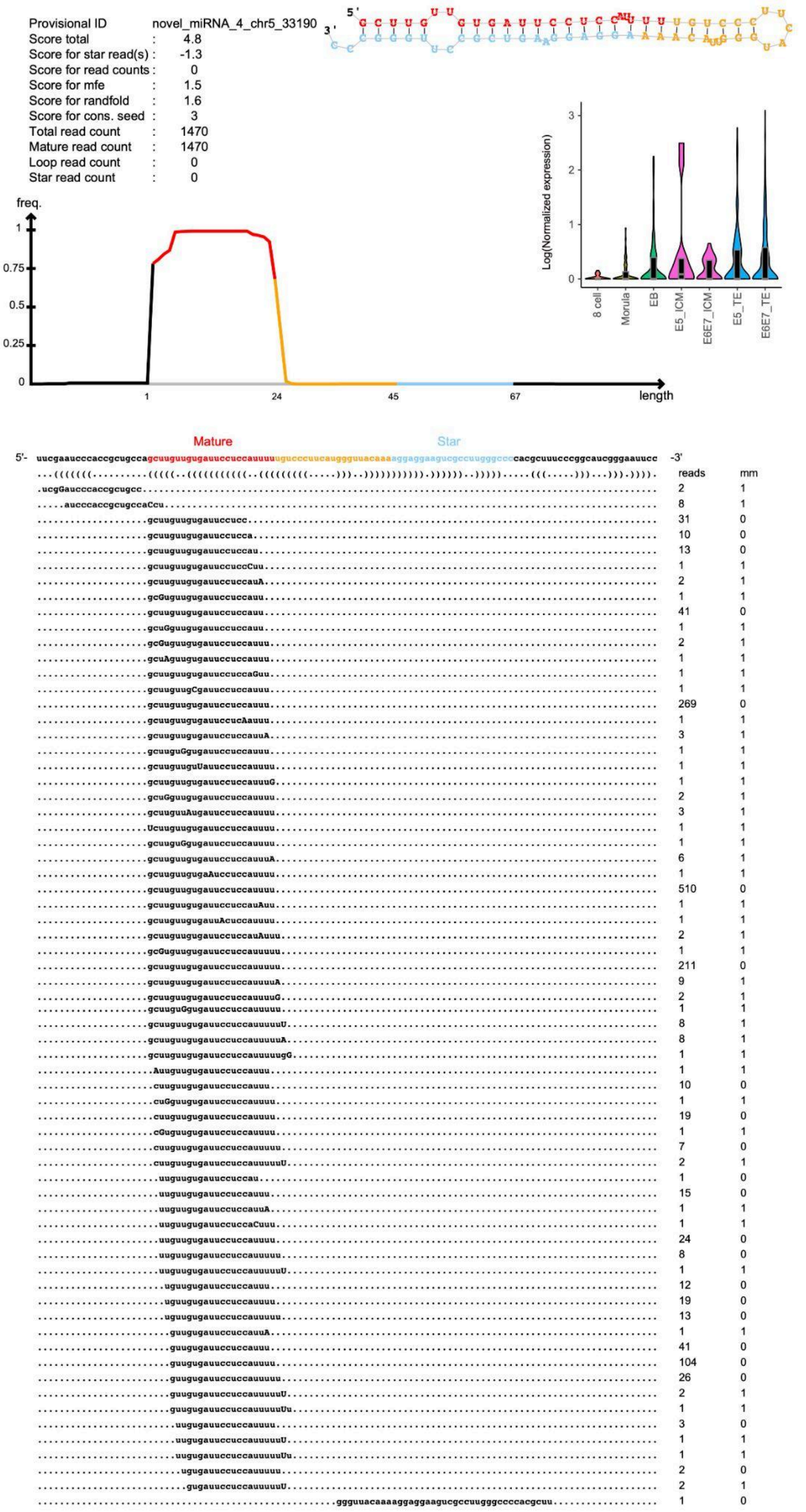

Supplementary Fig. 13 miRDeep2 output and human embryonic expression for novel_miRNA_4_chr5_33190. E - embryonic day; ICM - inner cell mass; TE - trophectoderm; EB - early blastocyst.

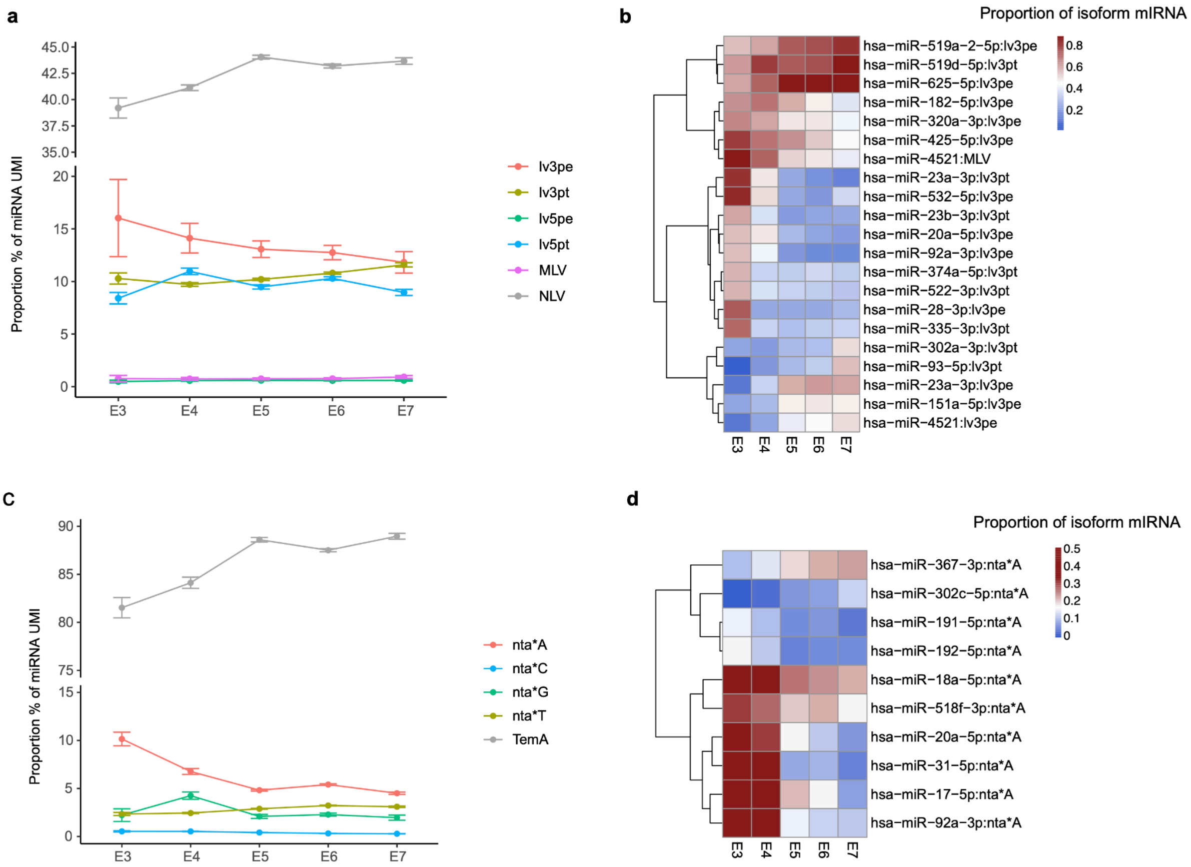

Supplementary Fig. 14 IsomiR expression dynamics through E3 to E7 embryo development. **a** Proportion of isomiR UMI with particular length variations. **b** Top 21 isomiRs and their expression changes across embryonic days (E)3-E7. **c** Proportion of isomiR UMI with particular non-templated additions. **d** Top 10 isomiRs with NTAs and their expression changes across development. Embryo and cell numbers are presented in Fig 1a.

Supplementary Table 1: Primers used for targeted miRNA qPCR

**Target**	**Forward Primer Sequence**	**Reverse Primer Sequence**
RT_primer	CAGGTCCAGTTTTTTTTTTTTTTTVN	N/A
hsa-miR-26a-5p	GCAGTTCAAGTAATCCAGGATAG	GGTCCAGTTTTTTTTTTTTTTTAGC
hsa-miR-146b-5p	GCAGTGAGAACTGAATTCCA	CCAGTTTTTTTTTTTTTTTCAGCCT
hsa-miR-182-5p	AGTTTGGCAATGGTAGAACTC	GTCCAGTTTTTTTTTTTTTTTAGTGTG
hsa-miR-27b-3p	GCAGTTCACAGTGGCTAAG	TCCAGTTTTTTTTTTTTTTTGCAGA
hsa-miR-23a-3p	AGATCACATTGCCAGGGAT	GGTCCAGTTTTTTTTTTTTTTTGGAA
hsa-miR-518b	CAAAGCGCTCCCCTTTAG	GTCCAGTTTTTTTTTTTTTTTACCTC
