# Supplementary material for: An atlas of small non-coding RNAs in human preimplantation development
Source: Nat Commun. 2024 Oct 5;15:8634. doi: 10.1038/s41467-024-52943-w (PMC11452719; doi:10.1038/s41467-024-52943-w)
Supplement: Supplementary file 3 — Description of Additional Supplementary Files [file 41467_2024_52943_MOESM3_ESM.pdf]

Description of Additional Supplementary Information

Supplementary Data 1: Cell metadata.

Supplementary Data 2: miRNA markers.

Supplementary Data 3: Average expression of non-coding RNAs (ncRNAs).

Supplementary Data 4: Differentially expressed miRNAs.

Supplementary Data 5: Trajectory analysis of miRNA gene targets.

Supplementary Data 6: Differentially expressed miRNAs between ICM and TE lineages.

Supplementary Data 7: Differential expression of genes in mouse embryos treated with miR-381 mimic.

Supplementary Data 8: Novel miRNA analysis.
