# Supplementary material for: An atlas of small non-coding RNAs in human preimplantation development
Source: Nat Commun. 2024 Oct 5;15:8634. doi: 10.1038/s41467-024-52943-w (PMC11452719; doi:10.1038/s41467-024-52943-w)
Supplement: Supplementary file 12 — Reporting Summary [file 41467_2024_52943_MOESM12_ESM.pdf]

nature portfolio

Corresponding author(s): Sophie Petropoulos

Last updated by author(s): Sep 4, 2024

Reporting Summary

Nature Portfolio wishes to improve the reproducibility of the work that we publish. This form provides structure for consistency and transparency in reporting. For further information on Nature Portfolio policies, see our Editorial Policies and the Editorial Policy Checklist.

Statistics

For all statistical analyses, confirm that the following items are present in the figure legend, table legend, main text, or Methods section.

n/a	Confirmed	
☐	☒	The exact sample size (*n*) for each experimental group/condition, given as a discrete number and unit of measurement
☐	☒	A statement on whether measurements were taken from distinct samples or whether the same sample was measured repeatedly
☐	☒	The statistical test(s) used AND whether they are one- or two-sided<br>*Only common tests should be described solely by name; describe more complex techniques in the Methods section.*
☒	☐	A description of all covariates tested
☐	☒	A description of any assumptions or corrections, such as tests of normality and adjustment for multiple comparisons
☐	☒	A full description of the statistical parameters including central tendency (e.g. means) or other basic estimates (e.g. regression coefficient) AND variation (e.g. standard deviation) or associated estimates of uncertainty (e.g. confidence intervals)
☐	☒	For null hypothesis testing, the test statistic (e.g. *F*, *t*, *r*) with confidence intervals, effect sizes, degrees of freedom and *P* value noted<br>*Give P values as exact values whenever suitable.*
☒	☐	For Bayesian analysis, information on the choice of priors and Markov chain Monte Carlo settings
☒	☐	For hierarchical and complex designs, identification of the appropriate level for tests and full reporting of outcomes
☐	☒	Estimates of effect sizes (e.g. Cohen's *d*, Pearson's *r*), indicating how they were calculated

Our web collection on statistics for biologists contains articles on many of the points above.

Software and code

Policy information about availability of computer code

Data collection

Datasets utilized in this study include the published Smart-seq2 preimplantation human embryonic dataset by Petropoulos et al., 2016 (E-MTAB-3929). Newly generated datasets(accession number GSE249713) include Small-seq and Co-seq data of preimplantation human embryos, as well as Smart-seq2 data from miR-381 mimic treated and control 8-cell and 32-cell mouse embryos.

Data analysis

Published software included:

FastQC (v0.11.9) was used to check reads quality.
STAR aligner (v2.5.1b) and RSEM (v1.3.0) were used to map Smart-seq2 data.
miRDeep2 (v2.0.1.2) was used to predict novel miRNAs.
umi_tools (v0.4.4), cutadapt (v1.17), and bowtie (v1.0.0) were used to map Small-seq data.
IGV (v2.11.9) was used to check read coverage for Small-seq reads.
The R package Seurat (v4.2.0) was used for processing Smart-seq2 data and Small-seq data.
The R packages DESeq (v1.38.0), scran (v1.14.6), and batchelor (v1.2.4) were used for normalization.
The R package slingshot (v2.6.0) was used to infer trajectory.
The R package TSCAN (v1.28.0) was used to infer miRNAs related to trajectory.
The R package pheatmap (v1.0.12) was used for visualization and k-means clustering.
The R package fgsea (v1.28.0) was used for gene set enrichment analysis.

Custom code is available at https://zenodo.org/records/12818575

April 2023

For manuscripts utilizing custom algorithms or software that are central to the research but not yet described in published literature, software must be made available to editors and reviewers. We strongly encourage code deposition in a community repository (e.g. GitHub). See the Nature Portfolio guidelines for submitting code & software for further information.

Data

Policy information about availability of data

All manuscripts must include a data availability statement. This statement should provide the following information, where applicable:

- Accession codes, unique identifiers, or web links for publicly available datasets
- A description of any restrictions on data availability
- For clinical datasets or third party data, please ensure that the statement adheres to our policy

Raw read sequencing files (FASTQ), along with unfiltered read count expression matrices, have been deposited in the Gene Expression Omnibus (GEO) database with accession number GSE249713 [https://www.ncbi.nlm.nih.gov/geo/query/acc.cgi?acc=GSE249713]. The expression matrices for the preimplantation embryo dataset used in this study were sourced from ArrayExpress, accession number E-MTAB-3929 [https://www.ebi.ac.uk/biostudies/arrayexpress/studies/E-MTAB-3929]. Source data for all graphs/figures are provided as a Source Data File.

Research involving human participants, their data, or biological material

Policy information about studies with human participants or human data. See also policy information about sex, gender (identity/presentation), and sexual orientation and race, ethnicity and racism.

Reporting on sex and gender	Embryo sex was not considered in this analysis since sex determination based on small ncRNA expression is not feasible. Of note, we did not observe any segregation of cells within lineage or developmental time to suggest a stratification of data by sex. As such, for all downstream experiments, cells from male and female preimplantation embryos were grouped.
Reporting on race, ethnicity, or other socially relevant groupings	Maternal and paternal ethnicities were not considered as embryos utilized were anonymized.
Population characteristics	Female patient age ranged from 26-45, with an average of 36.6 years. Additional patient-specific covariates were not collected for the study.
Recruitment	Supernumerary human embryos donated to research by patients who have completed their fertility care were utilised.
Ethics oversight	The study was approved by the University of Toronto (protocol # 30251), Veritas IRB (protocol number 16580), Stockholm Regional Ethics Board (2018/691-31) and the Université de Montréal and Centre de Recherche du Centre Hospitalier de l'Université de Montréal regional ethics board (CERSES-20-107-R and 20.126, respectively)

Note that full information on the approval of the study protocol must also be provided in the manuscript.

Field-specific reporting

Please select the one below that is the best fit for your research. If you are not sure, read the appropriate sections before making your selection.

☒ Life sciences ☐ Behavioural & social sciences ☐ Ecological, evolutionary & environmental sciences

For a reference copy of the document with all sections, see nature.com/documents/nr-reporting-summary-flat.pdf

Life sciences study design

All studies must disclose on these points even when the disclosure is negative.

Sample size	A total of 95 human embryos were included in the study, which was determined by interim data analyses and limited by overall availability of donated embryos. No sample size calculation was performed, as there was insufficient data in the literature to estimate embryo cell to cell differences in small RNA content. The sample sizes provided in the manuscript are estimated to be sufficient, as they have resulted in a clear change in small RNA profile along embryonic time and lineage.
Data exclusions	Quality control for SmartSeq data included cells with the number of expressed genes (nGene) >750 and percentage of mitochondrial genes (percent.mito) <0.3. For small seq data, cell quality filtering was implemented based on three criteria: First, each cell required a minimum of 0.5 million sequenced reads. Second, the proportion of mitochondrial associated UMIs was required to be less than 25%. Third, the number of expressed miRNA molecules per cell was required to be more than 100.
Replication	Our data suggest that a sufficient number of single cells across 5 experimental batches were collected at each stage to generate unique clustering and reproducible lineage signatures.

Randomization	Only one experimental group was used for the human embryo small RNA profiling. For 2 cell mouse embryos , they were collected from mice and always arbitrarily and equally split between control and miRNA-mimic treated groups.
Blinding	Blinding was not possible as the scientist performing the experiment had to move the embryos into their treatment groups. However, blinding is not relevant to the study as there are no obvious differences in embryo characteristics at the 2 cell stage that would lead to biases in group allocation.

Reporting for specific materials, systems and methods

We require information from authors about some types of materials, experimental systems and methods used in many studies. Here, indicate whether each material, system or method listed is relevant to your study. If you are not sure if a list item applies to your research, read the appropriate section before selecting a response.

Materials & experimental systems

n/a	Involved in the study
☐	☒ Antibodies
☐	☒ Eukaryotic cell lines
☒	☐ Palaeontology and archaeology
☐	☒ Animals and other organisms
☒	☐ Clinical data
☒	☐ Dual use research of concern
☒	☐ Plants

Methods

n/a	Involved in the study
☒	☐ ChIP-seq
☒	☐ Flow cytometry
☒	☐ MRI-based neuroimaging

Antibodies

Antibodies used	anti-Sox2 (eBioscience™, clone: Btjce, cat # 14981182, lot # 2493179, 1:100), anti-Cdx2 (BioGenex, cat # MU392A-5UC, lot # MU392A0820, 1:250), anti-Tead1 (Cell Signaling, clone: D9X2L, cat # 12292, lot # 4, 1:100).
Validation	On the manufacturer websites of all the antibodies used, antibodies were confirmed to be reactive in mouse and suitable for immunofluorescence/immunocytochemistry. Validation that antibodies worked was based on the correct expression patterns in the ICM and TE lineages. For example, Cdx2 is known to be found exclusively in outer TE cells of mouse blastocysts while Sox2 is known to be expressed exclusively in inner cells. Patterns of Tead1 expression matched those found in a prior study by Nishioka et al., 2008: https://www.sciencedirect.com/science/article/pii/S0925477307001979

Eukaryotic cell lines

Policy information about cell lines and Sex and Gender in Research

Cell line source(s)	Healthy Control Human iPSC Line, Female, SCTi003-A, STEMCELL Technologies
Authentication	iPSC state was confirmed by qRT-PCR assessment of markers of naïve pluripotency (DPPA5, KLF4), primed state (OTX2, ZIC2), and general stem cell pluripotency (NANOG, POU5F1, SOX2). Additional cells were confirmed by low-pass NGS to be euploid.
Mycoplasma contamination	All cell lines tested negative for mycoplasma contamination
Commonly misidentified lines (See ICLAC register)	No commonly misidentified cell lines were used in the study

Animals and other research organisms

Policy information about studies involving animals; ARRIVE guidelines recommended for reporting animal research, and Sex and Gender in Research

Laboratory animals	Mouse, C57BL/6NCrl (Charles River Laboratories) x DBA/2NHsd (Inotiv/Envigo) F1 mice, 6-8 weeks old. All mouse experiments were approved by the Centre de Recherche du Centre Hospitalier de l'Universite de Montreal (CRCHUM) Comité Institutionnel de Protection des Animaux du CHUM (CIPA) under the protocol number IP21005SPs. Mice were maintained in individually ventilated cages (up to 5 animals/cage) at 22±2°C and 40-60% humidity in 12 h light/dark cycle with lights on from 6:30am-6:30pm with ad libitum access to food and water.
Wild animals	*Provide details on animals observed in or captured in the field; report species and age where possible. Describe how animals were caught and transported and what happened to captive animals after the study (if killed, explain why and describe method; if released, say where and when) OR state that the study did not involve wild animals.*
Reporting on sex	*Indicate if findings apply to only one sex; describe whether sex was considered in study design, methods used for assigning sex. Provide data disaggregated for sex where this information has been collected in the source data as appropriate; provide overall*

	numbers in this Reporting Summary. Please state if this information has not been collected. Report sex-based analyses where performed, justify reasons for lack of sex-based analysis.
Field-collected samples	*For laboratory work with field-collected samples, describe all relevant parameters such as housing, maintenance, temperature, photoperiod and end-of-experiment protocol OR state that the study did not involve samples collected from the field.*
Ethics oversight	2023-10655, ip22026SPs

Note that full information on the approval of the study protocol must also be provided in the manuscript.

Plants

Seed stocks	*Report on the source of all seed stocks or other plant material used. If applicable, state the seed stock centre and catalogue number. If plant specimens were collected from the field, describe the collection location, date and sampling procedures.*
Novel plant genotypes	*Describe the methods by which all novel plant genotypes were produced. This includes those generated by transgenic approaches, gene editing, chemical/radiation-based mutagenesis and hybridization. For transgenic lines, describe the transformation method, the number of independent lines analyzed and the generation upon which experiments were performed. For gene-edited lines, describe the editor used, the endogenous sequence targeted for editing, the targeting guide RNA sequence (if applicable) and how the editor was applied.*
Authentication	*Describe any authentication procedures for each seed stock used or novel genotype generated. Describe any experiments used to assess the effect of a mutation and, where applicable, how potential secondary effects (e.g. second site T-DNA insertions, mosiacism, off-target gene editing) were examined.*
